# Vitamin C induces specific demethylation of H3K9me2 in mouse embryonic stem cells via Kdm3a/b

**DOI:** 10.1186/s13072-017-0143-3

**Published:** 2017-07-12

**Authors:** Kevin T. Ebata, Kathryn Mesh, Shichong Liu, Misha Bilenky, Alexander Fekete, Michael G. Acker, Martin Hirst, Benjamin A. Garcia, Miguel Ramalho-Santos

**Affiliations:** 1 Eli and Edythe Broad Center of Regeneration Medicine and Stem Cell Research, University of California, San Francisco, San Francisco, CA USA; 2 Epigenetics Program, Department of Biochemistry and Biophysics, Perelman School of Medicine, University of Pennsylvania, Philadelphia, PA USA; 3 Canada’s Michael Smith Genome Sciences Centre, BC Cancer Agency, Vancouver, BC Canada; 4 Novartis Institutes for Biomedical Research, Cambridge, MA USA; 5 Department of Microbiology and Immunology, Centre for High-Throughput Biology, University of British Columbia, Vancouver, BC Canada

**Keywords:** Vitamin C, Histone methylation, Histone lysine demethylase, Epigenetics, Embryonic stem cells

## Abstract

**Background:**

Histone methylation patterns regulate gene expression and are highly dynamic during development. The erasure of histone methylation is carried out by histone demethylase enzymes. We had previously shown that vitamin C enhances the activity of Tet enzymes in embryonic stem (ES) cells, leading to DNA demethylation and activation of germline genes.

**Results:**

We report here that vitamin C induces a remarkably specific demethylation of histone H3 lysine 9 dimethylation (H3K9me2) in naïve ES cells. Vitamin C treatment reduces global levels of H3K9me2, but not other histone methylation marks analyzed, as measured by western blot, immunofluorescence and mass spectrometry. Vitamin C leads to widespread loss of H3K9me2 at large chromosomal domains as well as gene promoters and repeat elements. Vitamin C-induced loss of H3K9me2 occurs rapidly within 24 h and is reversible. Importantly, we found that the histone demethylases Kdm3a and Kdm3b are required for vitamin C-induced demethylation of H3K9me2. Moreover, we show that vitamin C-induced Kdm3a/b-mediated H3K9me2 demethylation and Tet-mediated DNA demethylation are independent processes at specific loci. Lastly, we document Kdm3a/b are partially required for the upregulation of germline genes by vitamin C.

**Conclusions:**

These results reveal a specific role for vitamin C in histone demethylation in ES cells and document that DNA methylation and H3K9me2 cooperate to silence germline genes in pluripotent cells.

**Electronic supplementary material:**

The online version of this article (doi:10.1186/s13072-017-0143-3) contains supplementary material, which is available to authorized users.

## Background

Epigenetic information encoded in chromatin is crucial for cell identity and differentiation [1]. One major layer of chromatin-level regulation is histone methylation. In particular, histone lysine residues can be modified by mono-methylation (me1), dimethylation (me2), or tri-methylation (me3). Histone methyltransferases deposit methyl groups, which can then be recognized by reader proteins that modulate gene expression [2]. Depending on the reader proteins recruited to specific methylated histone residues, the corresponding genes may be repressed or activated. For example, H3K4me3 is associated with the recruitment of factors that promote gene activation, whereas H3K9me2/3 is associated with recruitment of repressive factors. In turn, histone methyl groups are removed by histone demethylases, most of which belong to the family of Fe(II)- and 2-oxoglutarate-dependent dioxygenases [2, 3]. The balance of the activity of histone methylases and demethylases determines the overall histone methylation patterns within a cell. Shifts in this balance underlie the extensive remodeling of histone methylation patterns that occurs during development.

The essential nutrient vitamin C has historically been described as a co-factor of collagen prolyl hydroxylases, the prototypical members of the family of Fe(II)- and 2-oxoglutarate-dependent dioxygenases, by recycling Fe(III) to Fe(II) [4]. The more recent discoveries that enzymes involved in DNA and histone demethylation are also Fe(II)- and 2-oxoglutarate-dependent dioxygenases [5] raises the interesting possibility that epigenetic programs may be modulated by diet [6]. In support of this notion, we and others reported that vitamin C enhances the activity of Tet enzymes, which are also Fe(II)- and 2-oxoglutarate-dependent dioxygenases, leading to widespread DNA demethylation and a blastocyst-like state in Embryonic Stem (ES) cells [7, 8].

Several studies have explored DNA methylation and histone mark patterning in ES cells during the transition from formative or primed (hypermethylated) to naïve (hypomethylated) state [9–12]. This transition can be modeled in vitro by switching ES cells maintained in serum containing media to a serum-free media supplemented with GSK3β and ERK1/2 inhibitors (2i) [9, 10]. In the presence of 2i, ES cells show a gradual global decrease in DNA methylation and concurrent reduction of H3K9me2 while maintaining H3K9me3 [11, 12]. Additionally, the distribution of H3K27me3 is globally redistributed with reduced levels at promoters in 2i [9] and accumulation at transposons after conversion to 2i + vitamin C [12]. Together, these studies identified a global reprogramming in DNA and histone methylation patterns during the primed to naïve state. However, the specific impact of vitamin C on histone methylation patterns and the potential interplay with DNA methylation and gene expression in ES cells maintained in the naïve (2i) state has not been investigated.

We report here that vitamin C leads to a remarkably specific and global reduction of histone H3 lysine 9 di-methylation (H3K9me2) in naïve mouse ES cells. The histone demethylases Kdm3a and Kdm3b are required for vitamin C-induced H3K9me2 demethylation, in a manner that is independent of Tet-mediated DNA demethylation. DNA methylation and H3K9me2 cooperated to repress germline gene expression in ES cells. These results highlight that histone methylation patterns are not indiscriminately sensitive to vitamin C. Moreover, our findings uncover a specific role for vitamin C in the epigenetic program of pluripotent cells, with implications for the regulation of germline development.

## Results

### Vitamin C induces a specific loss of H3K9me2 in ES cells

**Fig. 1:**
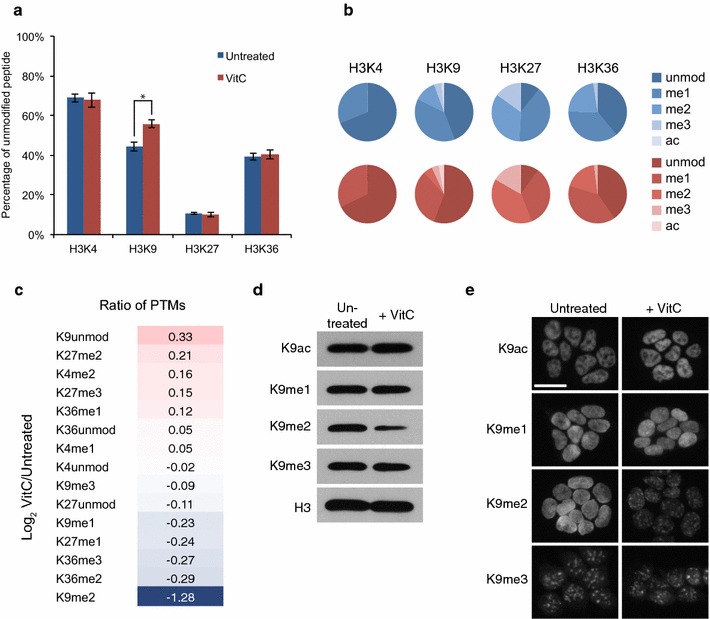
Vitamin C leads to a global reduction in H3K9me2 in ES cells. **a** Percentage of peptide with unmodified K4, K9, K27 or K36 residues on histone H3 from histone mass spectrometry performed on biological triplicates of untreated and vitamin C-treated ES cells. The percentage of unmodified H3K9 is significantly increased with vitamin C treatment. *Asterisk* represents *P* < 0.05 by *t* test. **b** Pie charts displaying the prevalence of various PTMs on histone H3 K4, K9, K27 and K36 residues in untreated (*blue*) and vitamin C-treated (*red*) ES cells. **c** Log_2_ ratio of indicated H3 PTMs in vitamin C-treated versus untreated ES cells. H3K9me2 shows the largest fold change and is decreased following vitamin C treatment. Unmodified H3K9 shows the largest increase. **d** Western blot for H3K9 PTMs in untreated and vitamin C-treated ES cells. **e** Immunofluorescence for H3K9 PTMs in untreated and vitamin C-treated ES cells. *Scale bar* represents 20 μm

We used mass spectrometry to perform an unbiased quantitative analysis of H3 N-terminal post-translational modifications (PTMs) in naïve mouse ES cells with or without vitamin C treatment for 72 h. Analysis of the percentages of unmodified H3K4, H3K9, H3K27, or H3K36 revealed that only unmodified H3K9 increased significantly in vitamin C-treated ES cells (Fig. 1a). Interestingly, there is a concomitant reduction in H3K9me2, but not H3K9me3, upon vitamin C treatment (Fig. 1b, c). H3K9me2 is reduced about 2.6-fold in vitamin C-treated ES cells, whereas there are minimal to no changes in other H3 PTM analyzed (Fig. 1c). Western blot of H3K9 PTMs confirmed a global reduction of H3K9me2 following vitamin C treatment, while H3K9me3, H3K9me1, and H3K9ac did not show significant changes (Fig. 1d). In agreement with the mass spectrometry data, we did not observe changes by Western blot in other H3 PTMs following vitamin C treatment (Additional file 1: Figure S1A). The lack of changes in H3K9me1 suggests that vitamin C promotes demethylation of both methyl groups of H3K9me2, leading to the increase in unmodified H3K9. Alternatively, vitamin C may promote demethylation of H3K9me2 to H3K9me1 and of H3K9me1 to H3K9me0 on short time scales such that overall H3K9me1 levels are not changed. Immunofluorescence for H3K9 PTMs showed an overall decrease in H3K9me2 staining intensity with vitamin C treatment as well as a change from diffuse nuclear staining to a punctate staining pattern that correspond to Dapi-dense heterochromatin (Fig. 1e; Additional file 1: Figure S1B). Residual H3K9me2 at condensed heterochromatin may be insensitive to vitamin C because it is less accessible to histone demethylases, and/or because it is a transient step on the way to H3K9me3.

The histone methyltransferase G9a and its binding partner G9a-like protein (GLP) can generate H3K9me2, but not H3K9me3 [13]. We therefore compared H3K9me2 levels in vitamin C-treated ES cells to *G9a* ^−/−^ and *GLP* ^−*/*−^ ES cells. These mutant ES cells have almost undetectable levels of H3K9me2, a much more profound loss of this mark than that induced by vitamin C treatment (Additional file 2: Figure S2A). Consistent with a previous study [14], H3K9me2 in *G9a* ^−/−^ and *GLP* ^−/−^ ES cells is present in a punctate pattern similar to vitamin C treatment (Additional file 2: Figure S2B), and this minimal residual H3K9me2 in *G9a* ^−/−^ and *GLP* ^−/−^ ES cells is further reduced by vitamin C (Additional file 2: Figure S2A). Overall, these data suggest that vitamin C induces partial loss of H3K9me2 generated by G9a/GLP.

### H3K9me2 is reduced at large chromosomal domains and gene promoters in ES cells treated with vitamin C

**Fig. 2:**
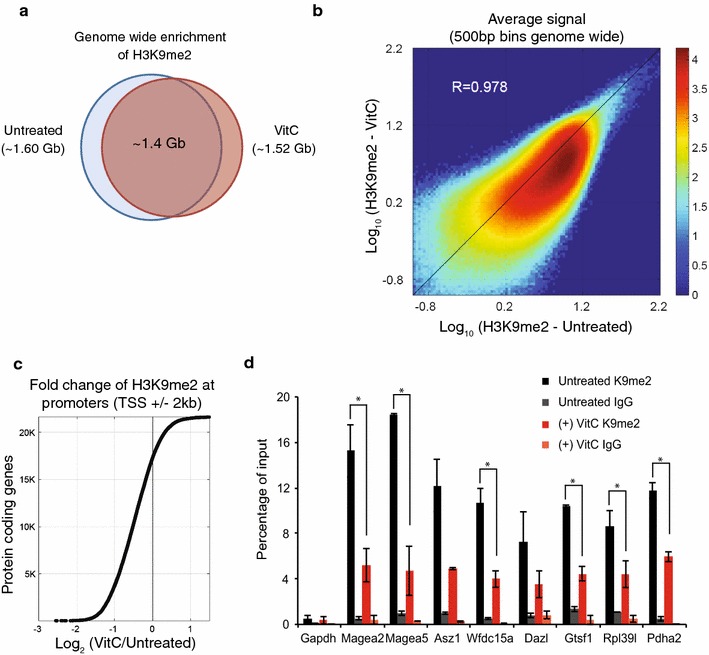
H3K9me2 is reduced globally across the genome including at gene promoters in ES cells treated with vitamin C. **a** ChIP-seq for H3K9me2 in ES cells treated with and without vitamin C was used to identify genomic regions enriched for H3K9me2. *Venn diagram* shows overlap of regions enriched for H3K9me2 with and without vitamin C treatment. **b** H3K9me2 signal genome-wide was plotted comparing untreated and vitamin C-treated cells. The H3K9me2 signal is reduced globally with vitamin C treatment despite an overall high correlation between the samples (Pearson correlation of ~0.98). **c** Sorted fold change for average H3K9me2 signal at gene promoters (TSS ± 2 kb). Most gene promoters display a reduction in H3K9me2 in vitamin C-treated ES cells. **d** ChIP-qPCR for H3K9me2 in ES cells ± vitamin C at gene promoters. ChIP for IgG was performed as a negative control. The *Gapdh* promoter is a negative control region devoid of H3K9me2. Data are mean ± SD. *Asterisks* represent *P* < 0.05 by *t* test

We next examined the effect of vitamin C treatment on the genome-wide distribution of H3K9me2 at a finer resolution using chromatin immunoprecipitation (ChIP) followed by next-generation sequencing (ChIP-seq). A rescaling factor was applied to correct for greater sequencing depth in the H3K9me2 data from vitamin C-treated ES cells (Additional file 3: Figure S3, see “Methods” section). The overall distribution of H3K9me2 is not affected with vitamin C treatment (Fig. 2a), but the level of H3K9me2 signal is reduced genome-wide (Fig. 2b), consistent with the western blot, Immunofluorescence and mass spectrometry data. Importantly, we found that gene promoters display a generalized reduction in H3K9me2 signal in vitamin C-treated ES cells (Fig. 2c).

To validate the ChIP-seq data, ChIP-qPCR for H3K9me2 was performed at promoters of germline genes that are de-repressed in ES cells following vitamin C treatment [7]. There is a two to threefold decrease in H3K9me2 in vitamin C-treated ES cells compared to untreated ES cells across all genes analyzed (Fig. 2d). A similar pattern is observed at repeat elements known to be marked by H3K9me2 (Additional file 4: Figure S4). Specificity of the ChIP was confirmed by immunoprecipitation with anti-IgG antibody as well as a primer set for the promoter of *Gapdh*, which is devoid of H3K9me2 (Fig. 2d). Taken together, these results indicate that vitamin C induces a widespread loss of H3K9me2 in ES cells at large chromosomal regions, repeat elements and promoters of repressed germline genes.

### Kdm3a and Kdm3b mediate vitamin C-induced loss of H3K9me2

**Fig. 3:**
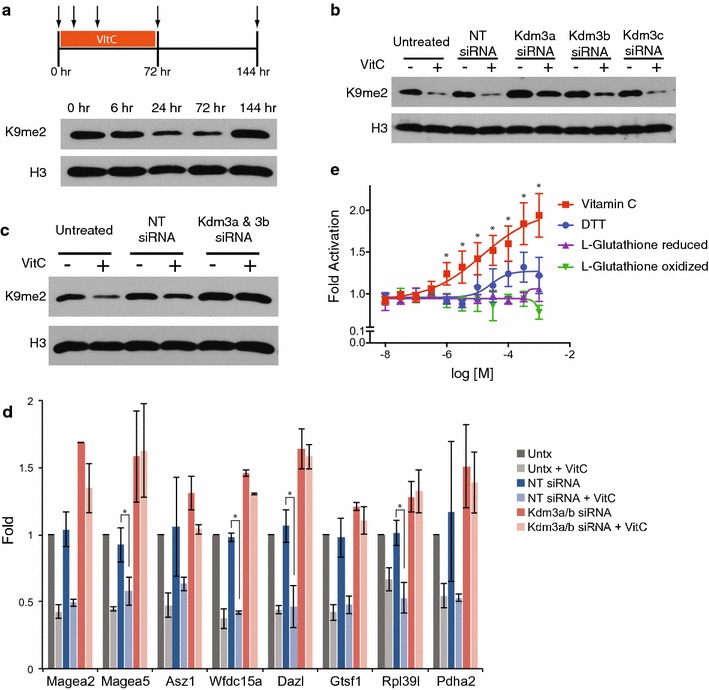
Kdm3a and Kdm3b mediate vitamin C-induced loss of H3K9me2. **a** Kinetics and reversibility of H3K9me2 loss following vitamin C treatment. H3K9me2 levels were measured by western blot at 6, 24, and 72 h following vitamin C treatment. Vitamin C was then removed, and H3K9me2 levels were measured 72 h later (144 h). **b** Western blot for H3K9me2 in ES cells ± vitamin C with siRNA knockdown of Kdm3 family enzymes or a non-targeting control. **c** Western blot for H3K9me2 in ES cells ± vitamin C with double knockdown of Kdm3a/3b or non-targeting control. **d** ChIP-qPCR for H3K9me2 at gene promoters in ES cells ± vitamin C with double knockdown of Kdm3a/3b or non-targeting control. Data are presented as fold change relative to the untreated control. Data are mean ± SD. *Asterisks* represent *P* < 0.05 by *t* test. **e** In vitro activity of recombinant KDM3A toward demethylation of a synthetic H3K9me1 peptide, in the presence of vitamin C, DTT or glutathione (see “Methods” section for details). Data are mean ± SD. *Asterisks* are *P* < 0.05 by *t* test for vitamin C compared to the oxidized form of glutathione

We next assessed the kinetics and reversibility of H3K9me2 loss upon vitamin C treatment. H3K9me2 is reduced after 24 h of vitamin C treatment, with no further reduction by 72 h (Fig. 3a). The loss of H3K9me2 is reversible and returns to baseline levels 72 h after vitamin C withdrawal (Fig. 3a). We therefore explored whether vitamin C may lead to reversible loss of H3K9me2 by shifting the balance of methylation/demethylation toward demethylation. Enzymes of the Kdm3 family are responsible for demethylating H3K9me1 and H3K9me2 [15]. There are three Kdm3 family members: *Kdm3a*, *Kdm3b*, and *Kdm3c* (also known as *Jmjd1a*, *Jmjd1b*, and *Jmjd1c*, respectively), although Kdm3c may not have catalytic activity [16]. Importantly, there is no change in the expression of Kdm3 family members following vitamin C treatment (Additional file 5: Figure S5A). We tested which Kdm3 family members might be involved in vitamin C-induced H3K9me2 demethylation, using RNAi. Knockdown efficiency and specificity was confirmed by qRT-PCR (Additional file 5: Figure S5B). Knockdown of Kdm3a or Kdm3b, but not Kdm3c, attenuated vitamin C-induced demethylation of H3K9me2 (Fig. 3b). Double knockdown of Kdm3a and Kdm3b completely abolished vitamin C-induced demethylation of H3K9me2 (Fig. 3c). ChIP-qPCR for H3K9me2 confirmed that Kdm3a/3b are required for vitamin C-induced loss of H3K9me2 (Fig. 3d). Moreover, we found that vitamin C enhances the in vitro activity of recombinant human KDM3A in demethylating a peptide at H3K9 (Fig. 3e; Additional file 6: Figure S6). These results indicate that vitamin C acts via Kdm3a/b enzymes to promote demethylation of H3K9me2.

### Interaction between vitamin C-induced changes in 5-methylcytosine/5-hydroxymethylcytosine and vitamin C-induced reduction in H3K9me2

**Fig. 4:**
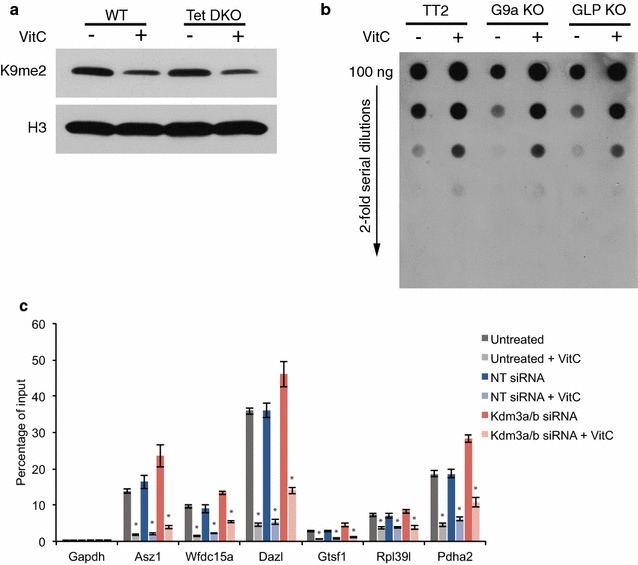
Interaction between vitamin C-induced increase in 5-hydroxymethylation and vitamin C-induced loss of H3K9me2. **a** Western blot for H3K9me2 in wild-type and Tet1/2 double knockout (Tet DKO) ES cells ± vitamin C. Vitamin C-induced loss of H3K9me2 still occurs in Tet DKO ES cells that do not undergo vitamin C-induced increase in 5-hmC. **b** Dot blot for 5-hydroxymethylcytosine (5-hmC) in wild-type parental TT2, G9a knockout, and GLP knockout ES cells ± vitamin C. Vitamin C-induced increase in 5-hmC still occurs in G9a and GLP knockout ES cells depleted of H3K9me2. **c** meDIP-qPCR on ES cells ± vitamin C that were untreated, treated with a non-targeting (NT) siRNA, or treated with siRNAs against Kdm3a and Kdm3b. Vitamin C-induced DNA demethylation occurs in the absence of Kdm3a/b and loss of H3K9me2. Data are mean ± SD. *Asterisks* represent *P* < 0.05 by *t* test

Vitamin C has previously been shown to enhance Tet activity in ES cells leading to an increase in 5-hydroxymethylcytosine (5-hmC) and DNA demethylation at gene promoters [7, 8]. There is extensive cross-talk between DNA methylation and histone methylation [17]. DNA demethylation could conceivably impact chromatin accessibility and affect the ability of H3K9me2 demethylases to act. We therefore sought to determine the interaction between vitamin C-induced changes in 5-hmC/5-mC and vitamin C-induced reduction in H3K9me2. Tet1/2 double knockout (Tet DKO) ES cells, which do not increase 5-hmC or demethylate promoters in response to vitamin C, still display a global loss of H3K9me2 following vitamin C treatment (Fig. 4a). Thus, vitamin C-induced loss of H3K9me2 can occur independently of vitamin C-induced Tet-mediated deposition of hydroxymethylation. Additionally, global 5-hmC is increased following vitamin C treatment in *G9a* ^−/−^ and *GLP* ^−/−^ ES cells to levels comparable to those in parental wild-type ES cells (Fig. 4b). Finally, we focused on germline genes that lose H3K9me2 (Fig. 2d), lose DNA methylation and are induced [7] in response to vitamin C. Kdm3a/b double knockdown ES cells have a similar extent of vitamin C-induced DNA demethylation as non-targeting siRNA-transfected ES cells; however, they appear to have slightly elevated baseline DNA methylation levels (Fig. 4c). These data indicate that vitamin C-induced DNA demethylation can occur independently of H3K9me2 demethylation at germline genes sensitive to vitamin C levels.

### Kdm3a/3b are partially required for vitamin C-induced upregulation of germline genes in ES cells

**Fig. 5:**
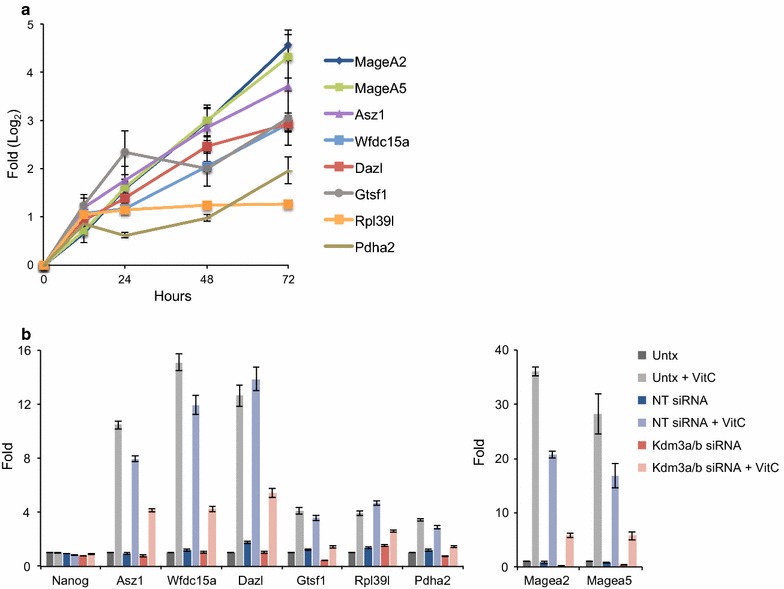
Kdm3a and Kdm3b are partially required for upregulation of genes following vitamin C treatment. **a** Kinetics of gene upregulation following vitamin C treatment. Log_2_ fold change in gene expression was evaluated by qRT-PCR at 12, 24, 48, and 72 h following vitamin C treatment. **b** Fold change in gene expression following vitamin C treatment with siRNA knockdown of Kdm3a/3b or non-targeting control. Vitamin C-induced upregulation of genes is attenuated with Kdm3a/3b knockdown

DNA methylation and H3K9me2 are both considered repressive marks that inhibit gene expression when present at gene promoters. We previously showed that vitamin C treatment in ES cells leads to upregulation of germline genes and that this upregulation is partially Tet-dependent [7]. Our time-course experiments indicate that loss of H3K9me2 occurs after 24 h (Fig. 3a), whereas DNA demethylation occurs more slowly and takes 72 h [7]. We sought to determine whether loss of H3K9me2 contributes to the activation of germline genes by vitamin C. First, we analyzed the kinetics of germline gene induction following vitamin C treatment, between 12 and 72 h, and found that vitamin C-induced gene induction occurs progressively over this time period (Fig. 5a). Next, we found that double knockdown of Kdm3a/b attenuates vitamin C-induced germline gene induction (Fig. 5b). Overall, these data suggest that DNA methylation and H3K9me2 are partially redundant mechanisms of repression of germline genes.

## Discussion

We report here that vitamin C leads to the specific and widespread loss of H3K9me2 in ES cells, via a mechanism that requires the Kdm3a/3b histone demethylases and is independent of Tet-mediated DNA demethylation. Vitamin C has also been shown to increase the efficiency of reprogramming of somatic cells to the induced pluripotent stem cell state, and this effect is likely mediated by both Tet enzymes and histone demethylases [18, 19].

The remarkable stability of other histone marks in this study raises the possibility that not all histone demethylases are equally dependent on vitamin C. Although our analysis of global histone marks by Western and mass spectrometry showed a specific loss of H3K9me2, it does not exclude the possibility that vitamin C enhances the activity of other histone demethylases at a local level. Interestingly, in contrast to the loss of H3K9me2 we observed in ES cells, vitamin C was shown to promote loss of H3K36me2/3 via KDM2A/2B in mouse embryonic fibroblasts. These results suggest that different cell types, varying in many factors including expression levels of different histone demethylases, may affect the extent to which histone methylation patterns are sensitive by vitamin C [20]. It will be important to study the effect of vitamin C on histone methylation patterns in other cell types and investigate a potential structural basis for the differential sensitivity of histone demethylases to vitamin C.

Given the genome-wide loss of H3K9me2 (this study) and DNA methylation [7] induced by vitamin C, it is interesting that the transcriptome of ES cells is largely unchanged, with the exception of a subset of germline-associated genes [7]. It is possible that other genetic programs remain silenced by other repressive mechanisms, such as H3K27me3 [21], which we show here is largely insensitive to vitamin C, and/or the lack of transcriptional activators. We found that vitamin C-induced loss of H3K9me2 is independent of Tet-mediated DNA demethylation at specific germline genes and that both H3K9me2 and DNA methylation contribute to their repression in ES cells. Our results indicate that pluripotent cells have the ability to express germline genes, but that these are repressed by H3K9me2 and DNA methylation. Interestingly, both H3K9me2 and DNA methylation are detected at high levels in pluripotent epiblast cells in vivo and are extensively erased during germline (but not soma) development [22–25]. Moreover, methods to generate germline-like cells from pluripotent stem cells in vitro involve culture in the presence of knockout serum replacement (KSR), which contains vitamin C [26, 27]. It will be of interest to assess whether vitamin C regulates H3K9me2, DNA methylation or gene expression during germline development, in vivo or in vitro. In addition, our results raise the possibility that vitamin C availability may regulate other processes where H3K9me2 has been shown to play a role, such as in hematopoietic lineage commitment [28], brain development [29] or adult metabolism [30].

## Conclusions

In this study, we investigated the role of vitamin C in regulating histone methylation in naïve ES cells. We performed comprehensive profiling of histone methylation by mass spectrometry following vitamin C treatment and found a specific reduction in H3K9me2. The distribution pattern of H3K9me2 was not changed by vitamin C, instead levels were reduced throughout the genome, including at gene promoters. Mechanistic studies showed that vitamin C-induced loss of H3K9me2 is via Kdm3a/b enzymes. Vitamin C reduces both H3K9me2 and DNA methylation independently at germline gene promoters, allowing expression of these genes in ES cells.

## Methods

### ES cell lines and cell culture

*Oct4*-GiP, TT2, *G9a* ^−/−^, *GLP* ^−/−^, V6.5, and *Tet1* ^−/−^ *Tet2* ^−/−^ ES cells were used in this study. ES cells were cultured on tissue culture plates coated with 0.1% gelatin in 2i medium, which consists of N2B27 base medium [31] supplemented with the MEK inhibitor, PD0325901 (1 μM, Stemgent), the GSK3β inhibitor, CHIR99021 (3 μM, Selleck Chemicals), and with ESGRO leukemia inhibitory factor (LIF) at 1000 U/ml (Millipore). Vitamin C (L-ascorbic acid 2-phosphate, Sigma, A8960) was added on day 1 after seeding at 100 μg/ml. Medium was replaced daily.

### Immunofluorescence

Cultured cells were fixed in 4% paraformaldehyde for 15 min, washed 3× with PBS, and blocked with blocking solution (PBS + 0.5% Tween20 + 5% FBS) for 1 h at room temperature (RT). Primary antibodies were diluted in blocking solution and incubated with cells overnight at 4 °C. Primary antibodies included H3K9ac (1:1000, Active Motif, 39917), H3K9me1 (1:2000, Abcam, ab9045), H3K9me2 (1:1000, Abcam, ab1220), H3K9me3 (1:1000, Diagenode, pAb-056-050). Cells were then washed for 5 min 3× in PBS and incubated with secondary antibodies in blocking solution for 2 h at RT. Secondary antibodies included 594-conjugated donkey anti-mouse (1:1000, Life Technologies, A21203) and 594-conjugated donkey anti-rabbit (1:1000, Life Technologies, A21207). DAPI (1 μg/ml) was added with secondary antibodies. Cells were washed for 5 min 3× in PBS prior to imaging.

### Histone extraction for quantitative mass spectrometry and Western blotting

Core histones were extracted from cultured ES cells using the Histone Purification Mini Kit (Active Motif, 40026). Following extraction, core histones were precipitated in 4% perchloric acid overnight at 4 °C, washed, and resuspended in water.

### Western blot

Isolated histones were resolved on a 4–20% gradient TGX gel and transferred to PVDF membranes. Membranes were blocked in blocking solution (Li-Cor Odyssey Buffer diluted 1:1 with PBS) for 1 h at RT. Primary antibodies were diluted in blocking solution and incubated for 3 h at RT. Membranes were washed for 10 min 3× in TBST (TBS + 0.5% Tween) and incubated with secondary antibodies diluted in blocking solution for 2 h at RT. Membranes were washed for 10 min 3× in TBST and visualized with Pierce ECL Plus.

### Quantitative mass spectrometry (qMS)

Approximately 20 μg of extracted histones was resuspended in 30 μl of 100 mM ammonium bicarbonate, at pH 8.0. Chemical propionylation derivatization, digestion and desalting of histones followed by analysis by LC–MS and MS/MS were performed as described previously [32, 33]. In brief, purified peptides were loaded onto 75-μm-ID fused-silica capillary columns packed with 12 cm of C18 reversed-phase resin (Reprosil-pur 120 C18, aq-3 μm particles, Fisher Scientific). Peptides were separated using EASY-nLC nano-HPLC (Thermo Scientific, Odense, Denmark) and introduced into a hybrid linear quadrupole ion trap–Orbitrap mass spectrometer (ThermoElectron) and resolved with a gradient from 0 to 35% solvent *B* (*A* = 0.1% formic acid; *B* = 95% MeCN, 0.1% formic acid) over 30 min and from 34 to 100% solvent *B* in 20 min at a flow-rate of 250 nL/min. The Orbitrap was operated in data-dependent mode essentially as previously described [33]. Relative abundances of peptide species were calculated by chromatographic peak integration of full MS scans using EpiProfile [34]. Where necessary, peptide and PTM identity were verified by manual inspection of MS/MS spectra.

### Chromatin immunoprecipitation

Isolated ES cells were resuspended in PBS and crosslinked with 1% formaldehyde for 5 min at RT. Crosslinking was quenched with 125 mM glycine. Crosslinked material was sonicated on a Covaris sonicator for 12 min at duty 5%, intensity 3, and bursts 200. ChIP was performed using the Diagenode LowCell# Kit with a mouse anti-H3K9me2 antibody (Abcam, ab1220). A mouse anti-IgG antibody (Abcam, ab18413) was used as control. Isolated DNA was used for qPCR analysis with the KAPA SYBR Fast ABI Prism qPCR kit on an Applied BioSystems 7900HT Sequence Detection System. Primer sequences are listed in Additional file 7: Table S1. For ChIP-seq isolated DNA was resuspended in an elution buffer (10 mM Tris–HCl pH 7.6, 200 mM NaCl, 5 mM EDTA, 0.5% SDS) with 10 μl RNAse and reverse-cross-linked at 65 °C for 4 h. Proteinase K was added and samples were incubated at 55 °C for 1 h. DNA was purified with QIAquick and quantified with a Qubit Fluorometer (Invitrogen). ChIP-seq libraries were constructed from immunoprecipitated DNA as described [35]. Libraries were sequenced using paired-end 100nt sequencing V3 chemistry on an HiSeq 2000 sequencer following the manufacturer’s protocols (Illumina, Hayward, CA.) using multiplex custom index adapters added during library construction to distinguish pooled samples.

### Dot blot analysis

Dot blot analysis was performed as previously described [7]. Briefly, isolated DNA was denatured and then serially diluted twofold. DNA samples were spotted on a nitrocellulose membrane using a Bio-Dot apparatus (Bio-Rad) and immunoblotted with rabbit anti-5-hydroxymethylcytosine polyclonal antibody (Active Motif, 1:5000) in Odyssey:PBS (Li-Cor). The membrane was washed and then incubated with HRP-conjugated goat anti-rabbit IgG (Abcam, 1:10,000) secondary antibody in Odyssey:PBS. The membrane was visualized by chemiluminescence with GE ECL Plus.

## 5mC DNA immunoprecipitation

DNA immunoprecipitation was performed using the Diagenode MagMeDIP as previously described [7]. Briefly, DNA was sonicated into short fragments with a Diagenode Bioruptor. Sonicated DNA (1 μg) was immunoprecipitated with 1 μg of mouse anti-5-methylcytosine monoclonal antibody (Active Motif, 1 μg/μl). Isolation of immunoprecipitated DNA was performed according to the kit instructions, and qPCR was performed in combination with the KAPA SYBR Fast ABI Prism qPCR kit on an Applied BioSystems 7900HT Sequence Detection System. Primer sequences are listed in Additional file 7: Table S1.

### qRT-PCR

Total RNA was isolated from cultured cells using Qiagen RNeasy with on-column DNase I treatment. cDNA was generated from 1 μg of RNA using random hexamers to prime the reaction. Quantitative RT-PCR was performed with the KAPA SYBR Fast ABI Prism qPCR kit on an Applied BioSystems 7900HT sequence detection system. Primer sequences are listed in Additional file 7: Table S1. The relative amount of each gene was normalized using two housekeeping genes (*L7* and *Ubb*), unless otherwise indicated.

### siRNA transfection

Oct4-GiP ES cells were transfected in suspension with Dharmacon siGENOME SMARTpool siRNAs (4 siRNAs per gene) against *Kdm3a*, *Kdm3b*, or *Kdm3c* at 50 nM and seeded into tissue culture plates (Day 0). Cells were re-transfected on day 2 followed by treatment with vitamin C for 24 or 72 h. For double knockdown of Kdm3a and Kdm3b, cells were transfected with 50 nM of both Kdm3a and Kdm3b siRNAs for a total final concentration of 100 nM. Dharmacon siGENOME non-targeting siRNA (NT2) was used as a control. Dharmafect reagent was used for transfections according to manufacturer’s instructions.

### KDM3a biochemical assay

The KDM3A assay was performed in 384-well black flat-bottom plates (Corning, Cat# 3654). Enzyme and substrate solutions were prepared in assay buffer consisting of 5 mM HEPES pH 7.0, 15 mM HEPES pH 7.5, 25 mM NaCl, 1 µM (or 1 mM) Alpha-ketoglutarate, 3.75 µM FeSO_4_, 0.014% Tween 20, 0.03% BSA. Dose–response concentrations of vitamin C (MP Biochemicals, 100769), DTT (Sigma, D0632) or glutathione (Affymetrix, 16315; Chem-Impex, 00158) were as indicated in the graphs. The KDM3A enzyme was produced in house [16]. H3K9me1 substrate was purchased from Perkin Elmer. Plates were covered and incubated for 30 min at room temperature. The reaction is stopped with detection buffer consisting of 100 mM HEPES pH 7.0, 800 mM KF and 0.2% BSA containing the detection reagents Eu-antiH3K9me0 (purchased from Perkin Elmer) and SA:APC (i.e., XL-665, purchased from Cisbio). The final concentrations of the various reagents were: KDM3A 2 nM, H3K9me1 500 nM, Eu-antiH3K9me0 0.75 nM, SA:APC 1.67 µg/ml. Plates were read in a Perkin Elmer Envision Plate Reader (ex/em 340/615 and 340/665).

### Bioinformatics

ChIP-seq raw sequences were examined for quality, sample swap and reagent contamination using custom in house scripts. Sequence reads were aligned to mm10 using BWA 0.5.7 and default parameters and assessed for overall quality using Findpeaks 3.1 [36]. For rescaling, we calculated average ChIP-seq coverage in 500 bp genomic bins. We considered ~150 bins with the highest signal in untreated data samples and calculated distributions of the fold change between vitamin C-treated and untreated data. These bins typically correspond to alignment artifacts and have extreme coverage not due to enrichment. They appear in both IP and control data sets with a very similar coverage profile, as validated by visual inspection of the majority of those locations in the UCSC genome browser. The alignment artifacts have a universal nature (mostly due to incomplete knowledge of genome) with the expectation that for similar cell types and the same read length and DNA fragment length distributions, the difference in the coverage for these ‘outliers’ will be a reflection of the differences in the sequencing depth. We observed that, as expected, for the input sample, the fold change for the total genomic coverage between vitamin C-treated and untreated data is in agreement with median fold change measured from ‘outliers’ bins. For the H3K9me2 signal, these twofold change values are very different, and for the ‘outliers’ the fold change is larger than a value of 1, suggesting that vitamin C-treated data had to be rescaled with a factor ~0.57 (Additional file 3: Figure S3). We verified that the value for the scaling factor depends only weakly on the exact threshold for choice of the outliers within a reasonable range. Using the rescaled data, the coverage profiles for DNA fragments corresponding to properly paired aligned reads were calculated and used to determine the average coverage in 500 bp bins genome wide (5,459,336 bins) and in the promoter regions (TSS ± 2 Kb) of the coding genes (21,958 genes, Ensembl v81).

## Additional files

**Additional file 1: Figure S1.**Evaluation of changes in H3 PTMs following vitamin C treatment. A) Western blot for several H3 PTMs in ES cells ± vitamin C. B) Immunofluorescence for H3K9me2 and corresponding DAPI staining in untreated and vitamin C-treated ES cells. Merged images show H3K9me2 in green and DAPI staining in red. H3K9me2 immunofluorescence is also shown in Fig. 1e. Scale bar represents 20 μm.

**Additional file 2: Figure S2.** Analysis of H3K9me2 in G9a and GLP knockout ES cells treated with vitamin C. A) Western blot for H3K9me2 in wild-type parental TT2, G9a knockout, and GLP knockout ES cells ± vitamin C. B) Immunofluorescence for H3K9me2 in GiP ES cells ± vitamin C and untreated wild-type TT2, G9a knockout, and GLP knockout ES cells. GiP ES cells treated with vitamin C show a H3K9me2 staining pattern that is similar to G9a and GLP knockout ES cells. Scale bar represents 20 μm.

**Additional file 3: Figure S3.** Differences in sequencing between untreated and vitamin C-treated H3K9me2 ChIP-seq samples. Average coverage in 500 bp bins was calculated for H3K9me2 ChIP-seq and DNA input samples. The top 150 bins with the highest signal in untreated samples were used to calculate distributions of the fold change between vitamin C-treated and untreated data. These bins with high signal typically correspond to alignment artifacts (see “Methods” section). As expected for the Input sample, the fold change for the total genomic coverage between vitamin C-treated and untreated data is in agreement with the median fold change measured for the outlier top 150 bins. For the H3K9me2 ChIP-seq samples, the difference between the twofold changes suggests that the vitamin C sequencing data has to be rescaled with a factor of ~0.57.

**Additional file 4: Figure S4.** Analysis of H3K9me2 at repetitive elements in ES cells treated with vitamin C. ChIP-qPCR for H3K9me2 in ES cells ± vitamin C at the repetitive element families indicated. ChIP for IgG was performed as a negative control. Data are mean ± SD. Asterisks represent *P* < 0.05 by *t* test.

**Additional file 5: Figure S5.** Effect of vitamin C treatment and siRNA knockdown on the expression of Kdm enzymes. A) Expression of Kdm family enzymes as a percentage of housekeeping gene in ES cells ± vitamin C. B) Gene expression levels of Kdm3a, Kdm3b, and Kdm3c following siRNA knockdown. Data are presented as fold change relative to the untreated control. A non-targeting (NT) siRNA was also used as a control. Each siRNA was applied in the presence or absence of vitamin C to show that vitamin C treatment does not affect knockdown efficiency.

**Additional file 6: Figure S6.** Effect of α-ketoglutarate on recombinant KDM3A activity with vitamin C, DTT and glutathione. In vitro activity of recombinant KDM3A toward demethylation of a synthetic H3K9me1 peptide, in the presence of vitamin C, DTT or glutathione, at 1 μM α-KG (see “Methods” section for details). At this lower concentration of α-KG, both vitamin C and DTT can enhance activity of KDM3A, but the effect of DTT saturates, whereas vitamin C does not. Data are mean ± SD. Asterisks represent *P* < 0.05 by t test for vitamin C compared to the oxidized form of Glutahione.

**Additional file 7: Table S1.** Primer list.
