# Supplementary material for: Vitamin C induces specific demethylation of H3K9me2 in mouse embryonic stem cells via Kdm3a/b
Source: Epigenetics Chromatin. 2017 Jul 12;10:36. doi: 10.1186/s13072-017-0143-3 (PMC5506665; doi:10.1186/s13072-017-0143-3)
Supplement: Supplementary file 1 — Additional file 1: Figure S1.Evaluation of changes in H3 PTMs following vitamin C treatment. A) Western blot for several H3 PTMs in ES cells ± vitamin C. B) Immunofluorescence for H3K9me2 and corresponding DAPI staining in untreated and vitamin C-treated ES cells. Merged images show H3K9me2 in green and DAPI staining in red. H3K9me2 immunofluorescence is also shown in Fig. 1e. Scale bar represents 20 μm. [file 13072_2017_143_MOESM1_ESM.pdf]

Figure S1

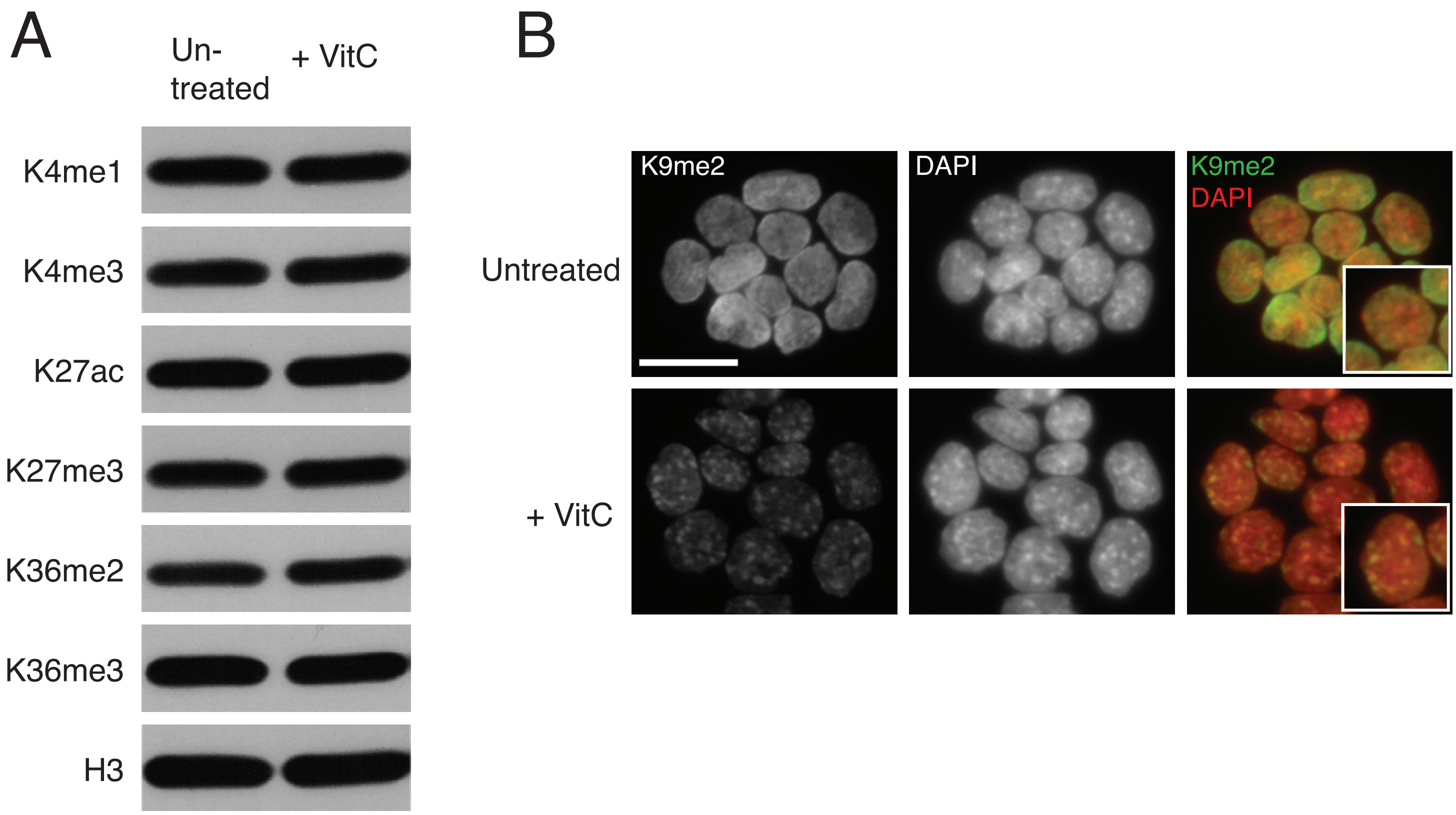

Figure S1. Evaluation of changes in H3 PTMs following vitamin C treatment.
A) Western blot for several H3 PTMs in ES cells +/- vitamin C. B) Immunofluorescence for H3K9me2 and corresponding DAPI staining in untreated and vitamin C-treated ES cells. Merged images show H3K9me2 in green and DAPI staining in red. H3K9me2 immunofluorescence also shown in Fig 1E. Scale bar represents 20μm.
