# Supplementary material for: Vitamin C induces specific demethylation of H3K9me2 in mouse embryonic stem cells via Kdm3a/b
Source: Epigenetics Chromatin. 2017 Jul 12;10:36. doi: 10.1186/s13072-017-0143-3 (PMC5506665; doi:10.1186/s13072-017-0143-3)
Supplement: Supplementary file 2 — Additional file 2: Figure S2. Analysis of H3K9me2 in G9a and GLP knockout ES cells treated with vitamin C. A) Western blot for H3K9me2 in wild-type parental TT2, G9a knockout, and GLP knockout ES cells ± vitamin C. B) Immunofluorescence for H3K9me2 in GiP ES cells ± vitamin C and untreated wild-type TT2, G9a knockout, and GLP knockout ES cells. GiP ES cells treated with vitamin C show a H3K9me2 staining pattern that is similar to G9a and GLP knockout ES cells. Scale bar represents 20 μm. [file 13072_2017_143_MOESM2_ESM.pdf]

Figure S2

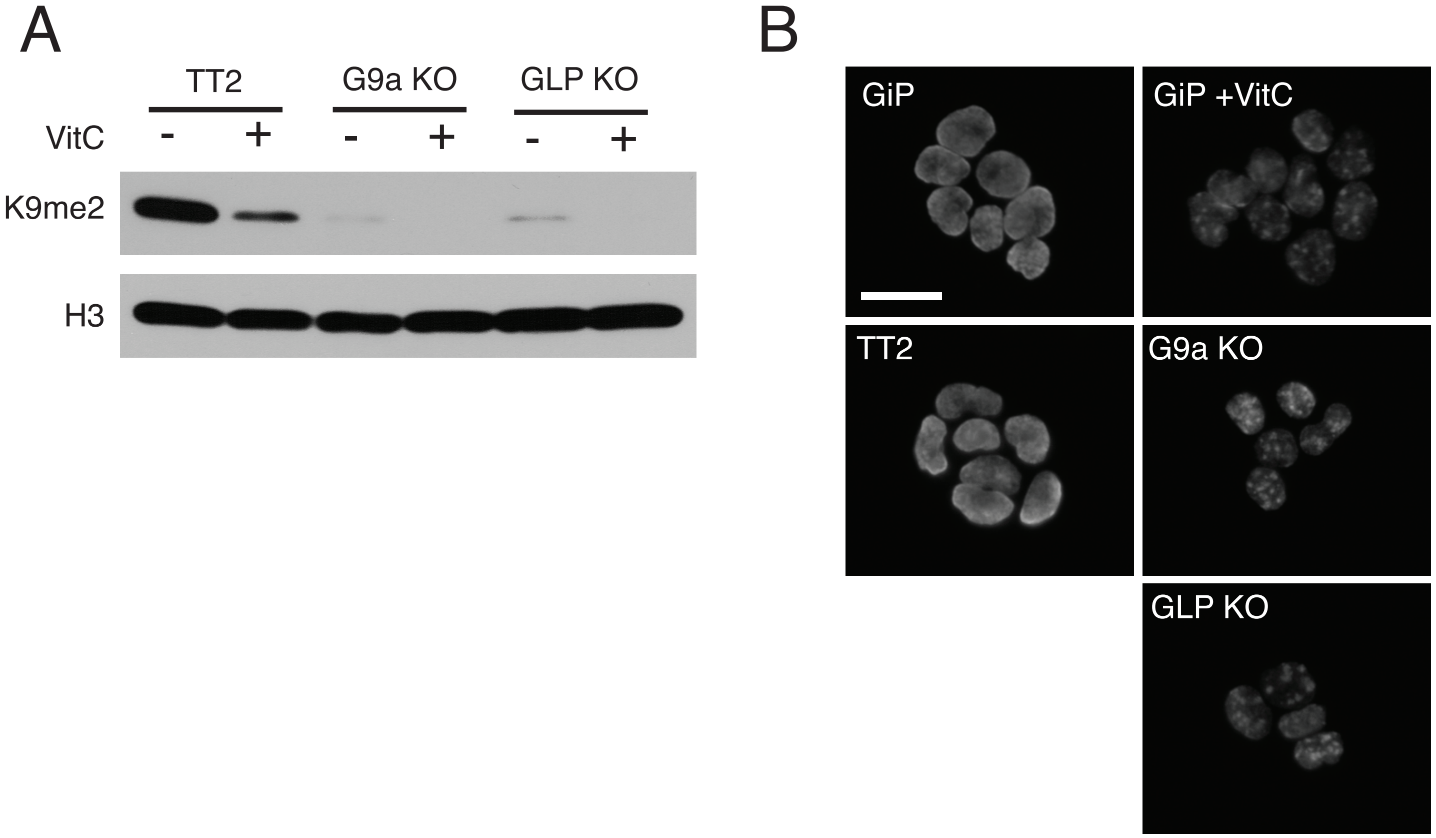

Figure S2. Analysis of H3K9me2 in G9a and GLP knockout ES cells treated with vitamin C.
A) Western blot for H3K9me2 in wildtype parental TT2, G9a knockout, and GLP knockout ES cells +/- vitamin C. B) Immunofluorescence for H3K9me2 in GiP ES cells +/- vitamin C and untreated wildtype TT2, G9a knockout, and GLP knockout ES cells. GiP ES cells treated with vitamin C show a H3K9me2 staining pattern that is similar to G9a and GLP knockout ES cells. Scale bar represents 20µm.
