# Supplementary material for: Vitamin C induces specific demethylation of H3K9me2 in mouse embryonic stem cells via Kdm3a/b
Source: Epigenetics Chromatin. 2017 Jul 12;10:36. doi: 10.1186/s13072-017-0143-3 (PMC5506665; doi:10.1186/s13072-017-0143-3)
Supplement: Supplementary file 3 — Additional file 3: Figure S3. Differences in sequencing between untreated and vitamin C-treated H3K9me2 ChIP-seq samples. Average coverage in 500 bp bins was calculated for H3K9me2 ChIP-seq and DNA input samples. The top 150 bins with the highest signal in untreated samples were used to calculate distributions of the fold change between vitamin C-treated and untreated data. These bins with high signal typically correspond to alignment artifacts (see “Methods” section). As expected for the Input sample, the fold change for the total genomic coverage between vitamin C-treated and untreated data is in agreement with the median fold change measured for the outlier top 150 bins. For the H3K9me2 ChIP-seq samples, the difference between the twofold changes suggests that the vitamin C sequencing data has to be rescaled with a factor of ~0.57. [file 13072_2017_143_MOESM3_ESM.pdf]

# Figure S3

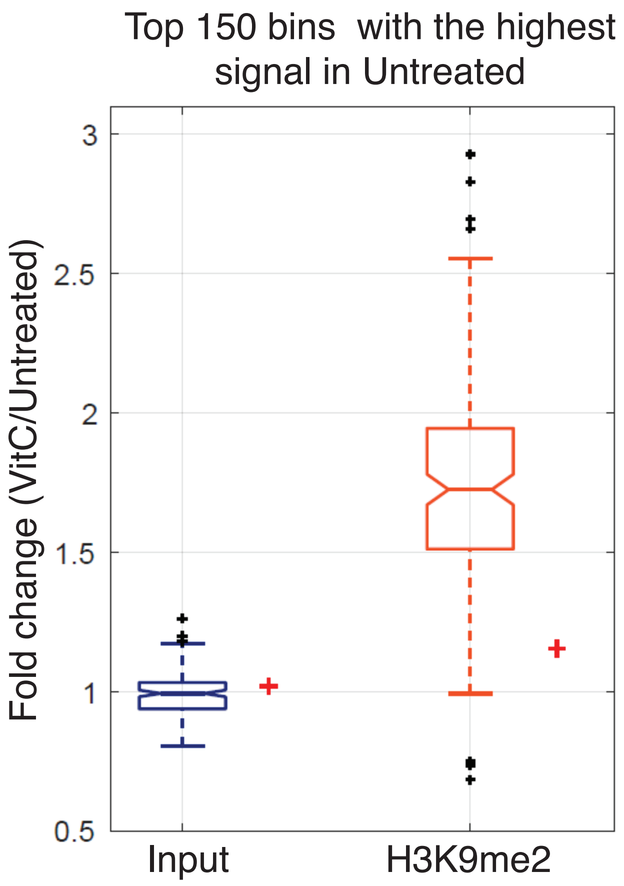


**Figure S3. Differences in sequencing between untreated and vitamin C-treated H3K9me2 ChIP-seq samples.**

Average coverage in 500bp bins were calculated for H3K9me2 ChIP-seq and DNA input samples. The top 150 bins with the highest signal in untreated samples were used to calculate distributions of the fold change between vitamin C-treated and untreated data. These bins with high signal typically correspond to alignment artifacts (see Methods). As expected for the Input sample, the fold change for the total genomic coverage between vitamin C-treated and untreated data is in agreement with the median fold change measured for the outlier top 150 bins. For the H3K9me2 ChIP-seq samples, the difference between the two fold changes suggests that the vitamin C sequencing data has to be rescaled with a factor of ~0.57.
