# Supplementary material for: Vitamin C induces specific demethylation of H3K9me2 in mouse embryonic stem cells via Kdm3a/b
Source: Epigenetics Chromatin. 2017 Jul 12;10:36. doi: 10.1186/s13072-017-0143-3 (PMC5506665; doi:10.1186/s13072-017-0143-3)
Supplement: Supplementary file 4 — Additional file 4: Figure S4. Analysis of H3K9me2 at repetitive elements in ES cells treated with vitamin C. ChIP-qPCR for H3K9me2 in ES cells ± vitamin C at the repetitive element families indicated. ChIP for IgG was performed as a negative control. Data are mean ± SD. Asterisks represent P < 0.05 by t test. [file 13072_2017_143_MOESM4_ESM.pdf]

Figure S4

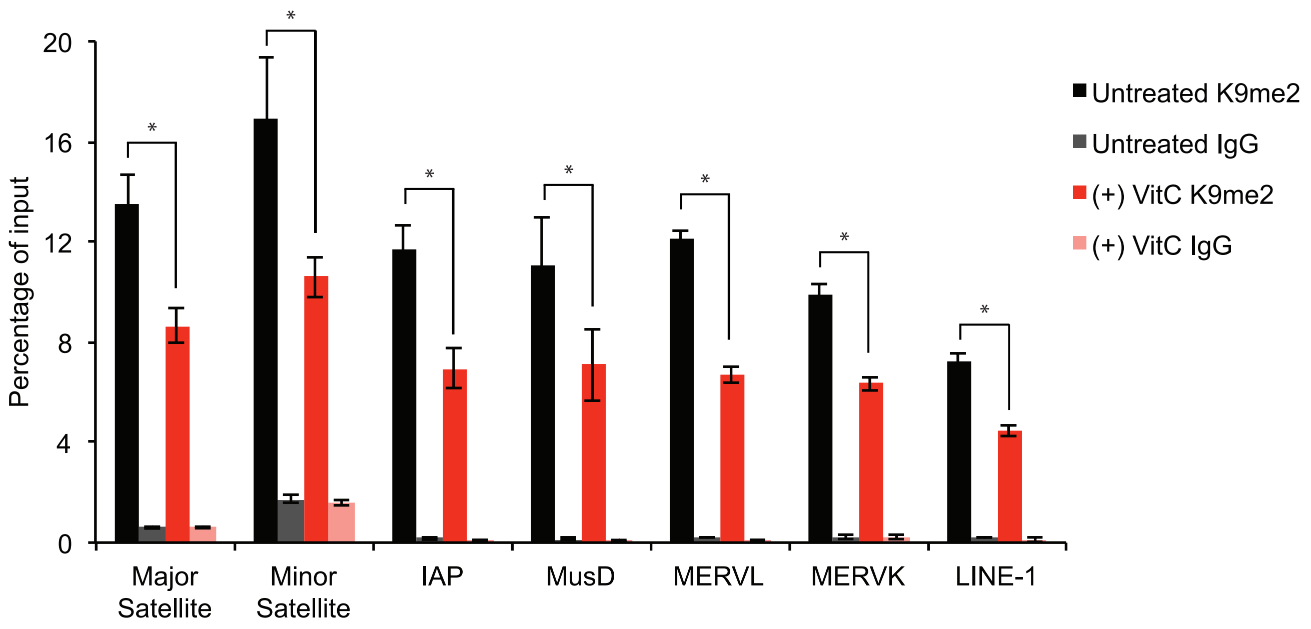

Figure S4. Analysis of H3K9me2 at repetitive elements in ES cells treated with vitamin C. ChIP-qPCR for H3K9me2 in ES cells +/- vitamin C at the repetitive element families indicated. ChIP for IgG was performed as a negative control. Data are means ± SD. Asterisks represent $P < 0.05$ by t-test.
