# Supplementary material for: Vitamin C induces specific demethylation of H3K9me2 in mouse embryonic stem cells via Kdm3a/b
Source: Epigenetics Chromatin. 2017 Jul 12;10:36. doi: 10.1186/s13072-017-0143-3 (PMC5506665; doi:10.1186/s13072-017-0143-3)
Supplement: Supplementary file 5 — Additional file 5: Figure S5. Effect of vitamin C treatment and siRNA knockdown on the expression of Kdm enzymes. A) Expression of Kdm family enzymes as a percentage of housekeeping gene in ES cells ± vitamin C. B) Gene expression levels of Kdm3a, Kdm3b, and Kdm3c following siRNA knockdown. Data are presented as fold change relative to the untreated control. A non-targeting (NT) siRNA was also used as a control. Each siRNA was applied in the presence or absence of vitamin C to show that vitamin C treatment does not affect knockdown efficiency. [file 13072_2017_143_MOESM5_ESM.pdf]

Figure S5

A

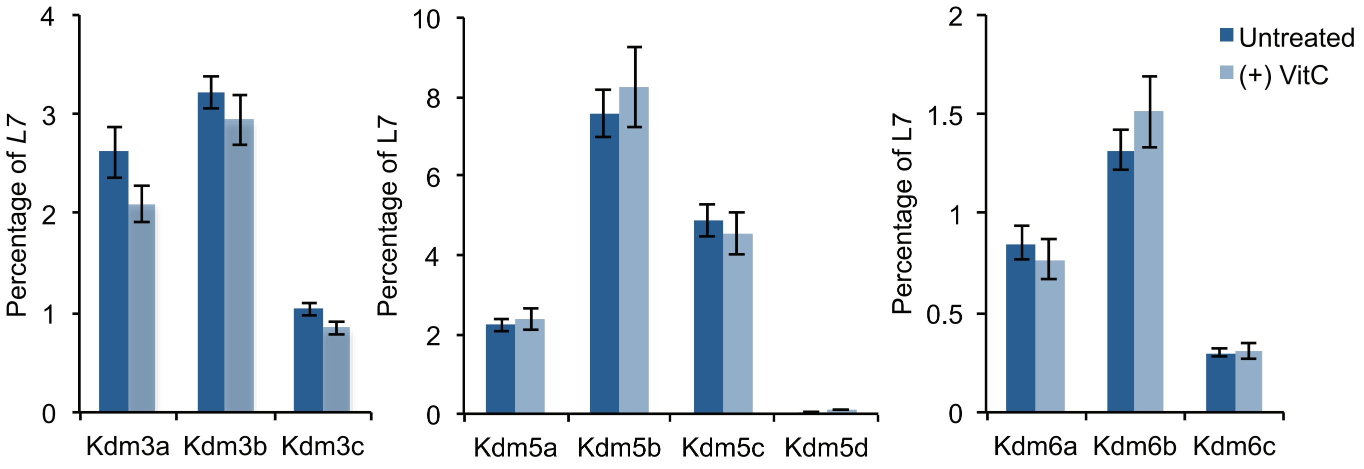

B

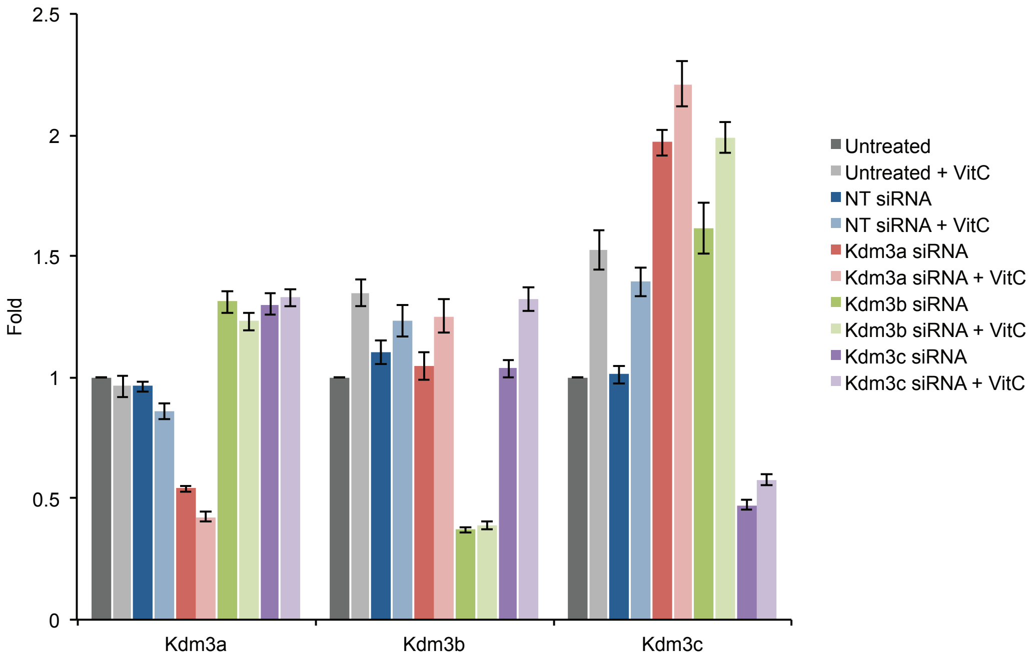

Figure S5. Effect of vitamin C treatment and siRNA knockdown on the expression of Kdm enzymes.

A) Expression of Kdm family enzymes as a percentage of housekeeping gene in ES cells +/- vitamin C. B) Gene expression levels of Kdm3a, Kdm3b, and Kdm3c following siRNA knockdown. Data is presented as fold change relative to the untreated control. A non-targeting (NT) siRNA was also used as a control. Each siRNA was applied in the presence or absence of vitamin C to show that vitamin C treatment does not affect knockdown efficiency.
