# Supplementary material for: Vitamin C induces specific demethylation of H3K9me2 in mouse embryonic stem cells via Kdm3a/b
Source: Epigenetics Chromatin. 2017 Jul 12;10:36. doi: 10.1186/s13072-017-0143-3 (PMC5506665; doi:10.1186/s13072-017-0143-3)
Supplement: Supplementary file 6 — Additional file 6: Figure S6. Effect of α-ketoglutarate on recombinant KDM3A activity with vitamin C, DTT and glutathione. In vitro activity of recombinant KDM3A toward demethylation of a synthetic H3K9me1 peptide, in the presence of vitamin C, DTT or glutathione, at 1 μM α-KG (see “Methods” section for details). At this lower concentration of α-KG, both vitamin C and DTT can enhance activity of KDM3A, but the effect of DTT saturates, whereas vitamin C does not. Data are mean ± SD. Asterisks represent P < 0.05 by t test for vitamin C compared to the oxidized form of Glutahione. [file 13072_2017_143_MOESM6_ESM.pdf]

Figure S6

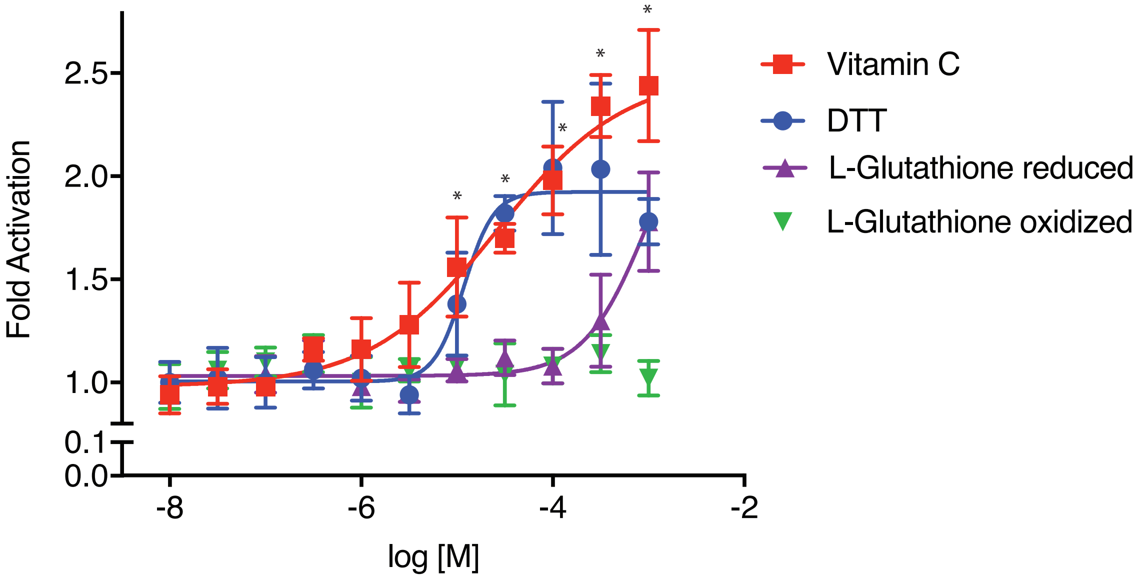

Figure S6. Effect of α-ketogluterate on recombinant KDM3A activity with vitamin C, DTT and glutathione.
In vitro activity of recombinant KDM3A towards demethylation of a synthetic H3K9me1 peptide, in the presence of vitamin C, DTT or Glutathione, at 1µM α-KG (see Methods for details). At this lower concentration of α-KG, both vitamin C and DTT can enhance activity of KDM3A, but the effect of DTT saturates, whereas vitamin C does not. Data are means ± SD. Asterisks are $P < 0.05$ by t-test for vitamin C compared to the oxidized form of Glutahione.
