# Supplementary material for: Vitamin C induces specific demethylation of H3K9me2 in mouse embryonic stem cells via Kdm3a/b
Source: Epigenetics Chromatin. 2017 Jul 12;10:36. doi: 10.1186/s13072-017-0143-3 (PMC5506665; doi:10.1186/s13072-017-0143-3)
Supplement: Supplementary file 7 — Additional file 7: Table S1. Primer list. [file 13072_2017_143_MOESM7_ESM.pdf]

Table S1

qRT-PCR	Forward(5'to3')	Reverse(5'to3')
Asz1	GAGTGGGCTTCTCCCAGAAA	GGTCATTTTCCCGCTCATTC
Dazl	CAACTGTTAACTACCACTGCAG	CAAGAGACCACTGTCTGTATGC
Gtsf1	GACTCCCTGGACCCTGAAAA	GCCAATTTGTTTGCGACATC
Kdm3a	GACCTGGGCCCCAAGATGTA	CCATGACATTGGCTGCATCAGA
Kdm3b	CGGTAGTGAGCGGAGATCCT	CAGCCGTCAAACTCCACAAA
Kdm3c	TTCTTGGATCTGTGACCAGCA	CTTATGCACACCGGAAACCA
Kdm4a	TCCCATCTATGGAGCAGACG	GGTCCAGGATGGTCTTCAGC
Kdm4b	CCATGGATGAATTCAGGGACTT	CCACTCCTTGGGTGGAATGA
Kdm4c	GACCATACAAAGCCCACTCCA	CAAAGTGGCTTATTGCCCTGT
Kdm4d	TGGATATGAAGAAGGCGCAAA	AGACTGGGGTGCAGCAGAAT
Kdm5a	TTGAAGATCCCTGTGGTGGA	GCGGCTACCCACTTTAGACC
Kdm5b	CCACCATTCGCTTGTGATGT	TTGCAATCTGGTCCAGGAAA
Kdm5c	ACCCACCTGGCAAAAACATT	TCATTATCAAATGGGCGTGTG
Kdm5d	CAGCCATCAAAATTCAGTTGC	GGCCTGCCCCATATATCTGTA
Kdm6a	TGAGCAACTTTTGCAGACAGAA	TCCTTGGTGGCCTTATCTCC
Kdm6b	GCCGGTCCTGCTACAGTTCT	CTCGCCTCCACCAGAGTCTT
L7	AGCGGATTGCCTTGACAGAT	AACTTGAAGGGCCACAGGAA
Magea2	GAGGTCTCCATGCTGTGCCA	TGGCGCCTAGTTCAAGTCCA
Magea5	GGTATCAAAGAGCTGAAACCCTGG	CGGATGTCTCCATGGTCTCCT
Nanog	AGGCTTTGGAGACAGTGAGGTGC	TACCCTCAAACTCCTGGTCCTTC
Pdha2	AAGGGCAGGTATTCGAAGCA	CTCGTTGGAGGTTCCCATTC
Rpl39l	TTTAAACTCGCCGGGAAGAG	TGTGGAATGGGACGATTTTG
Ubb	GAGAGGCTTTGTCCGGTTCG	TCTTCACGAAGATCTGCATTTTGAC
Wfdc15a	TGTGTGGAACCCTGGACAAC	GCCAATGCCGTCGTTATTTT

ChIP&DIP-qPCR	Forward(5'to3')	Reverse(5'to3')
Asz1	CCTCACTATCGCTGCTCTCG	CGCTCGCTCAAGCTCTGATA
Dazl	TACAAAATGCCCGCAGAAATAG	CCGGACTCAACCTTCTCAATG
Gapdh	CTCCGCGATTTTCACCTGGC	AATACGGACTGCAGCCCTCC
Gtsf1	TTCCTGTGACTGTGGCTTGC	GGAGGGTGAGCCAAAGAAAA
IAP	GCTCCTGAAGATGTAAGCAATAAAG	CTTCCTTGCGCCAGTCCCGAG
MageA2	GGAGCTAGGCAGGCTAAAGGT	TCTCCATGCTGTGCCAGTTG
MageA5	TGGCTTTCTTGCTACAAGGATTCT	AGTGGGAGGAGATTTTTGGGGA
Major satellite	GACGACTTGAAAAATGACGAAATC	CATATTCCAGGTCCTTCAGTGTGC
MERVK	TTCGCCTCTGCAATCAAGCTCTC	TCGCTCRTGCCTGAAGATGTTTC
MERVL	CTTCCATTCACAGCTGCGACTG	CTAGAACCACTCCTGGTACCAAC
MusD	CCCTTCCTTCATAACTGGTGTCGCA	TAGCATCTCTCTGCCATTCTTCAGG
Pdha2	TAAGATGGCTGCCGAGCTCC	GTGGCTTCCAGCTTGCTGAC
Rpl39l	TGCAGCTGACACTGGACACA	TCACTGTTGGTTTGGGGGCT
Wfdc15a	GGGAGGACGTTTGAATCTGC	GCACTTCCGTTTTCCTGACC
